# Inactivation of Foodborne Bacteria Biofilms by Aqueous and Gaseous Ozone

**DOI:** 10.3389/fmicb.2018.02024

**Published:** 2018-08-28

**Authors:** Marilena Marino, Michela Maifreni, Anna Baggio, Nadia Innocente

**Affiliations:** Dipartimento di Scienze Agroalimentari, Ambientali e Animali, Università degli Studi di Udine, Udine, Italy

**Keywords:** ozone, microbial biofilms, stainless steel, *Listeria monocytogenes*, *Pseudomonas fluorescens*, *Staphylococcus aureus*

## Abstract

In this study, the efficacy of treatments with ozone in water and gaseous ozone against attached cells and microbial biofilms of three foodborne species, *Pseudomonas fluorescens*, *Staphylococcus aureus*, and *Listeria monocytogenes*, was investigated. Biofilms formed on AISI 304 stainless steel coupons from a mixture of three strains (one reference and two wild strains) of each microbial species were subjected to three types of treatment for increasing times: (i) ozonized water (0.5 ppm) by immersion in static condition, (ii) ozonized water under flow conditions, and (iii) gaseous ozone at different concentrations (0.1–20 ppm). The Excel add-in GinaFit tool allowed to estimate the survival curves of attached cells and microbial biofilms, highlighting that, regardless of the treatment, the antimicrobial effect occurred in the first minutes of treatment, while by increasing contact times probably the residual biofilm population acquired greater resistance to ozonation. Treatment with aqueous ozone under static conditions resulted in an estimated viability reduction of 1.61–2.14 Log CFU/cm^2^ after 20 min, while reduction values were higher (3.26–5.23 Log CFU/cm^2^) for biofilms treated in dynamic conditions. *S. aureus* was the most sensitive species to aqueous ozone under dynamic conditions. With regard to the use of gaseous ozone, at low concentrations (up to 0.2 ppm), estimated inactivations of 2.01–2.46 Log CFU/cm^2^ were obtained after 60 min, while at the highest concentrations a complete inactivation (<10 CFU/cm^2^) of the biofilms of *L. monocytogenes* and the reduction of 5.51 and 4.72 Log CFU/cm^2^ of *P. fluorescens* and *S. aureus* respectively after 60 and 20 min were achieved. Considering the results, ozone in water form might be used in daily sanitation protocols at the end of the day or during process downtime, while gaseous ozone might be used for the treatment of confined spaces for longer times (e.g., overnight) and in the absence of personnel, to allow an eco-friendly control of microbial biofilms and consequently reduce the risk of cross-contamination in the food industry.

## Introduction

Infectious diseases induced by microorganisms are increasing in frequency worldwide and are one of the main illness causes all over the world. According to the latest report of the European Centre for Disease Prevention and Control, 1.1 million cases of notifiable infectious diseases were accounted in the EU in 2014 (ECDC, 2016). Despite the enormous advancement in processing technologies to assure food safety, contaminated food and water still continue to cause infectious diseases worldwide, and this is not just an underdeveloped world problem. For example, in Europe, in 2016, foodborne outbreaks (including waterborne outbreaks) caused 49,950 illnesses, 3,869 hospitalizations and 20 deaths (EFSA, 2017). As well as causing foodborne diseases, microorganisms can lead to significant economic losses due to spoilage both at the primary production level and the retail. In fact, the increased attention on food safety in the past decades has decreased the focus on the damage of food through spoilage, particularly in developed countries where food is abundant. It has been estimated that approximately 1.3 billion tons of food is lost or wasted globally per year, and microbial spoilage is one of the main causes of food loss or wastes worldwide along the entire food supply chain (Bräutigam et al., 2014; Thyberg and Tonjes, 2016).

The hygienic condition of surfaces and equipment used for food processing has a significant influence on the presence of microbial pathogens and spoilers in food products. If cleaning and sanitizing processes are not performed in the correct manner, residues of organic and inorganic substances could remain and create a suitable environment for biofilm development. Biofilms are made of microorganisms adhered to and growing on a surface and are a prevalent mode of growth for microorganisms: in this form, they are enclosed in extracellular polymeric substances (EPS) which protect cells against adverse environmental conditions, especially antimicrobials. Several food spoilage and pathogenic bacteria have been reported to attach and form biofilms on different food contact surfaces, and biofilms have become challenging in a wide range of food industries, such as dairy plants (Stellato et al., 2015), fish and seafood processing areas (Takahashi et al., 2009), meat and poultry processing (Liu et al., 2015), catering establishments (Marino et al., 2011) as well as fermented beverages plants (Maifreni et al., 2015). Major food pathogens, such as *Listeria monocytogenes*, *Staphylococcus aureus*, and *Escherichia coli*, can form biofilms and so become a significant threat to consumers’ health (Dourou et al., 2011; Ferreira et al., 2014). It has been demonstrated that the colonization of surfaces by microbial pathogens can lead to outbreaks linked to the consumption of fresh produce (Beuchat, 2002).

Conventional strategies to control biofilms are currently based on chemical disinfection. However, these methods are not always efficient and ecologically friendly. In fact, it has been showed that during routinary sanitization procedures bacteria can become less sensitive or even resistant to an antimicrobial compound following intermittent or continuous exposure to sub-lethal concentrations (To et al., 2002). Moreover, there are a lot of environmental and human health concerns related to the use of chemical sanitizers, which calls for more environmentally friendly alternatives. Therefore, novel means for biofilms’ control are constantly sought.

In the last decades, interest in ozone has expanded in food field, due to the growing consumers’ demand for “greener” food additives, the FDA’s approval of ozone directly added in foods and the increasing consciousness that ozonation represents an environmentally friendly technology. Use of ozone in food processing has been legally approved in North America, Australia, New Zealand, Japan, and several European countries (Tiwari and Rice, 2012). Ozonation is becoming widely accepted in the food context as an eco-friendly technology worldwide (O’Donnell et al., 2012). Ozone is an effective antimicrobial due to its oxidizing capacity that inactivates microorganisms by the progressive oxidation of cell components. The high instability and reactivity of ozone determine its antimicrobial properties because it rapidly degrades back to molecular oxygen without leaving toxic by-products, with the released free oxygen atom which causes oxidation (Güzel-Seydim et al., 2004). Ozonation has been successfully used in the food field to control bacterial counts in fruit and vegetable (Ölmez and Akbas, 2009), dairy (Segat et al., 2014; Marino et al., 2015), meat and seafood products (Gelman et al., 2005). It has been shown that ozone can be used to modify the chemical and physical properties of various macromolecules (Sankaran et al., 2008; An and King, 2009; Segat et al., 2015). The wide application of ozone in food field is only limited by health and safety aspects since it is a toxic compound that causes headaches, dry throat, and irritation to the respiratory system and even irreversible lethal effects at high concentrations can occur (Cullen and Norton, 2012). Therefore, efficacious systems for the detection and catalytic or thermal inactivation of ozone are required for the safety of workers in food-processing plants (Kim et al., 1999).

Ozone is commonly generated by photochemical method or corona discharge method. Another method is based on electrolysis, which allows the ozone to be dissolved *in situ* in the process water as soon as it is formed with the minimum amount of equipment (Chen et al., 2016). To the best of our knowledge, no bibliographic data are available on the use of ozone generated by electrolysis as an antimicrobial strategy.

Currently, the experimental data concerning the antimicrobial activity of ozone with respect to microbial biofilms in the food context are still quite limited, so the potential of the use of ozone in water and gaseous form in the control of cross-contamination is not completely clear. Moreover, the few published works have been carried out on biofilms formed by single strains coming from reference collections (Dosti et al., 2005; Di Ciccio et al., 2014; Saha et al., 2014). However, it is known that wild strains may be more resistant to antimicrobials than reference strains (Otero et al., 2014), so that recently the study protocols of the behavior of microorganisms in the food context underline the need to use wild strains too, isolated from the same or a similar food matrix. In fact, to account for differences in growth and survival among different strains of the same species, tests should be carried out with a cocktail of at least three strains, a reference strain and two wild strains (Jofré et al., 2009; Álvarez-Ordóñez et al., 2015). The aim of this study was to investigate the disinfection efficacy of aqueous and gaseous ozone on attached cells and biofilms against foodborne bacterial strains belonging to the species *Pseudomonas fluorescens*, *S. aureus*, and *L. monocytogenes*.

## Materials and Methods

### Microorganisms and Culture Conditions

To account for variation in growth and survival, three strains for each species were used as follows: *P. fluorescens*: (i) CECT 378^T^; (ii) L22, dairy isolate; and (iii) Ve096, vegetable processing plant isolate; *S. aureus*: (i) DSM 20231^T^; (ii) DIAL301, processed meat isolate; and (iii) La018, dairy plant isolate; and *L. monocytogenes*: (i) DSM 20600^T^; (ii) Lm5, dairy plant isolate; and (iii) Lm29, fish processing plant isolate. The species assignment was assessed by 16S rRNA gene amplification (Martino et al., 2013). Stock cultures of each strain were stored at -80°C in Brain Heart Infusion (BHI; Oxoid, Milan, Italy) supplemented with 30% (v:v) glycerol. Whenever required, stock cultures were subcultured overnight twice in BHI at 30°C (*P. fluorescens*) or 37°C (*S. aureus* and *L. monocytogenes*). Before each test, a separate cocktail of the overnight cultures (about 10^9^ CFU/mL) of each species was prepared by mixing equal portions (5 mL) of each strain.

### Biofilm Formation and Evaluation of Biofilm Viability

Multi-strain mono-species biofilms were grown on cylindrical stainless steel AISI 304 coupons (1.27-cm diameter, 0.3-cm depth; area 3.73 cm^2^) subjected to sonication at 40 kHz for 10 min (UltraSonic Bath LBS2, Falc Instruments, Treviglio, Italy) before each trial. To grow biofilms, 1 mL of each cocktail culture (prepared as previously reported) was used to inoculate 500 mL of Luria Bertani broth (LB; Oxoid, Milan, Italy) into the reactor vessel of CDC Biofilm Reactor (CBR; BioSurface Technologies, Bozeman, MT, United States), obtaining an initial count of about 5 × 10^6^ CFU/mL. The CBR was operated under batch phase and moderate agitation (125 rpm) during 48 h at 30°C (for *P. fluorescens*) or at 37°C (for *S. aureus* and *L. monocytogenes*) in a thermostatic chamber. After 5 min, 2 h, 4 h, 8 h, 24 h, and 48 h, the coupons were aseptically removed from the CBR and washed twice with 10 mL of sterile saline (0.9% NaCl) to remove loosely adherent cells. To detach biofilm cells, each coupon was then placed in a 50-mL sterile tube containing 3 mL of Maximum Recovery Diluent (MRD; Oxoid, Milan, Italy) with eight sterile glass beads (5.5 mm diameter) and agitated on a non-orbital shaker (Velp Scientifica, Monza, Italy) for 2 min at room temperature. The microbial suspension was serially diluted for viable counts by the spread plate technique on BHI agar plates, i.e., 0.1 mL of each suspension (or its decimal dilution) was put on duplicate plates. After incubation at 30°C or 37°C for 24–48 h and colony counting, data were expressed in log CFU/cm^2^ (threshold CFU value 8.3 CFU/cm^2^).

### Treatments With Aqueous Ozone

The aqueous ozone was used to treat 2-h (called from here on “attached cells”) and 48-h biofilms (called from here on “biofilms”). Aqueous ozone was generated directly in tap water using an electrolytic cell (Adept -75; Electrolytic Ozone Inc., Boston, MT). Tap water was conveyed through a peristaltic pump at a rate of 1 L/min to the electrolytic cell, which generated ozone at a concentration of about 0.5 mg/L. To measure the ozone level dissolved in water a colorimetric method was used (HI 38054; Hanna Instruments, Villafranca Padovana, PD, Italy).

The aqueous ozone was used in two different ways:

(i) static, i.e., coupons carrying attached cells or biofilms were washed twice with 10 mL of 0.9% NaCl (w/v) and placed individually in 50 mL Falcon tubes, which were immediately filled with ozonated water. The tubes were closed and the coupons left for 20 s, 40 s, 1 min, 3 min, 5 min, 10 min, and 20 min.
(ii) dynamic, i.e., coupons were maintained under a flow of ozonated water for 20 s, 40 s, 1 min, 3 min, 5 min, 10 min, and 20 min.

For each treatment, “untreated controls” were coupons aseptically removed from CDC-biofilm reactor after 2- or 48-h, and immediately analyzed for viable counts. In a preliminary step, coupons in two biological replicates and two technical repetitions were submitted to a dipping in tap water in 50 mL Falcon tubes for 20 min (to simulate static treatment), and a maintenance under a continuous flow (1 L/min) of tap water for 20 min (dynamic treatment). Viable counts were then evaluated and means submitted to *T*-test against means of coupons just after removing from CDC-Biofilm Reactor. In all cases means resulted similar (*p* > 0.05).

The treatments were carried out at room temperature (RT; 20°C). After each treatment, the cells were detached from coupons and submitted to viable count evaluation as previously reported.

### Evaluation of Ozone Decay in Water

The decay of ozone concentration in water in the static conditions was measured using a colorimetric method (as previously described). Fifty milliliter of ozonated water were placed in 50 mL Falcon tubes and left at RT. After 0, 1, 5, 10, and 20 min, the ozone concentration was measured. At least two tubes for each time were tested.

### Treatments With Gaseous Ozone

The gaseous ozone was used to treat biofilms. Coupons were washed as previously reported and placed individually in 60 mm Petri dishes and placed inside a treatment chamber connected to an ozone generator (AIRNow OG-36AN2K; O3 Technology, Brescia, Italy) and equipped with an ozone analyzer (BMT 964; Mes-stechnik GMBH, Villach, Austria). Treatments were carried out at concentrations of 0.1, 0.15, 0.2, 2, 5, and 20 ppm for exposure times of 2, 5, 7, 10, 20, 30, and 60 min. At the end of each treatment, coupons were evaluated for viable counts as previously described.

For each treatment, “untreated controls” were coupons aseptically removed from CDC-Biofilm Reactor after 48-h and immediately analyzed for viable counts. In a preliminary step, coupons in two biological replicates and two technical repetitions were maintained inside the treatment chamber with the ozone generator in “OFF” position for 60 min. Viable counts were then evaluated, and means submitted to *T*-test against means of coupons just after removing from CDC-Biofilm Reactor. In all cases means resulted similar (*p* > 0.05).

### Statistical Analysis

Each trial was carried out in at least two biological replicates, i.e., parallel measurements of biologically distinct samples (cocktail culture independently grown). For each biological replicate, viability was evaluated on two coupons (two technical repetitions). The means obtained from replicate tests were subjected to one-way analysis of variance (*p* < 0.05), preceded by the Levene test to verify the homogeneity of variance between means using the Statistics 8.0 (Statsoft software, Tulsa, OK, United States). Differences between the means were assessed using the Tukey’s HSD *post hoc* test.

The microbial population densities (CFU/cm^2^) observed in the biofilm formation experiments were log-transformed and modeled with the Baranyi and Roberts model (Baranyi and Roberts, 1994). The Excel add-in DMFit ver. 3.0 (Baranyi and Tamplin, 2004) was used to calculate the estimates of kinetic parameters, i.e., initial count (expressed in Log CFU/cm^2^), lag time (h), maximum growth rate (Log CFU/cm^2^/h) and maximum cell count (Log CFU/cm^2^).

To model the survival of the attached cells and biofilms to ozone treatments, Log (N/N_0_) values were calculated, where *N* = viable count and *N*_0_ = initial viable count (untreated controls). Log (*N*/*N*_0_) values were shaped through the GInaFIT tool ver. 1.7 (Geeraerd et al., 2005). The goodness-of-fit was assessed based on the coefficient of determination (*R*^2^) and the root mean squared error (RMSE).

## Results

### Adhesion Kinetics on Stainless Steel

**FIGURE 1:**
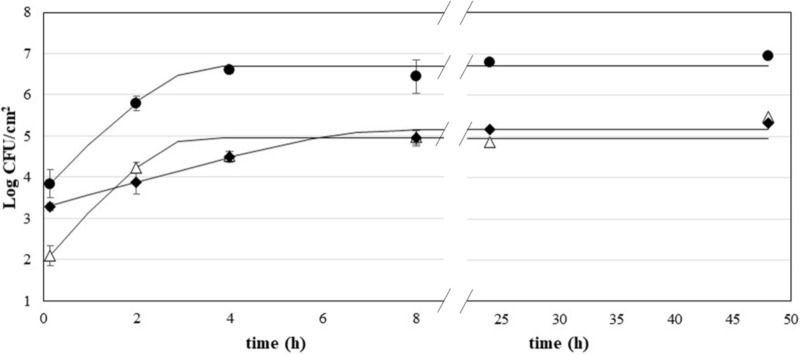
Growth of *P. fluorescens* (•), *S. aureus* (Δ), and *L. monocytogenes* (♦) on stainless steel; curves are fitted using the Baranyi and Roberts model.

**Figure 1** shows the adhesion kinetics of *P. fluorescens*, *S. aureus*, and *L. monocytogenes* on AISI 304 stainless steel coupons during a 48-h incubation at 30°C (*P. fluorescens*) or 37°C (*S. aureus* and *L. monocytogenes*). The kinetics were fitted according to the model of Baranyi and Roberts, with acceptable values of goodness-of-fit (**Table 1**). All species showed a rapid growth with no lag time. *P. fluorescens* was the fastest species in the very early adhesion phases, reaching viability values of 5.79 Log CFU/cm^2^ after 2 h of incubation. Instead, *S. aureus* and *L. monocytogenes* showed lower values (*p* < 0.05), 4.24 and 3.88 Log CFU/cm^2^, respectively. *P. fluorescens* biofilms had also the highest viability after 48 h (6.70 Log CFU/cm^2^). In contrast, *L. monocytogenes* and *S. aureus* showed significantly lower values of viability at the end of the incubation (*p* < 0.05). *L. monocytogenes* showed also the lowest value of maximum adhesion speed.

**Table 1:** Kinetic parameters of *P. fluorescens*, *S. aureus*, and *L. monocytogenes* estimated by fitting Baranyi and Roberts model to growth data on stainless steel.

	Initial cell count^∗^	Maximum growth rate^†^	Maximum cell count^§^	*R*^2^	RMSE
*Pseudomonas fluorescens*	3.84^a^ ± 0.22	0.98^a^ ± 0.16	6.70^a^ ± 0.11	0.96	0.22
*Staphylococcus aureus*	2.09^c^ ± 0.40	1.08^a^ ± 0.30	4.96^b^ ± 0.20	0.89	0.40
*Listeria monocytogenes*	3.29^b^ ± 0.14	0.30^b^ ± 0.05	5.16^b^ ± 0.09	0.96	0.15


*^∗^Log CFU/cm^2^; ^†^Log CFU/cm^2^/h; ^§^ Log CFU/cm^2^. For each column, means followed by different letters are significantly different (p < 0.05).*

### Ozone Decay in Water

**FIGURE 2:**
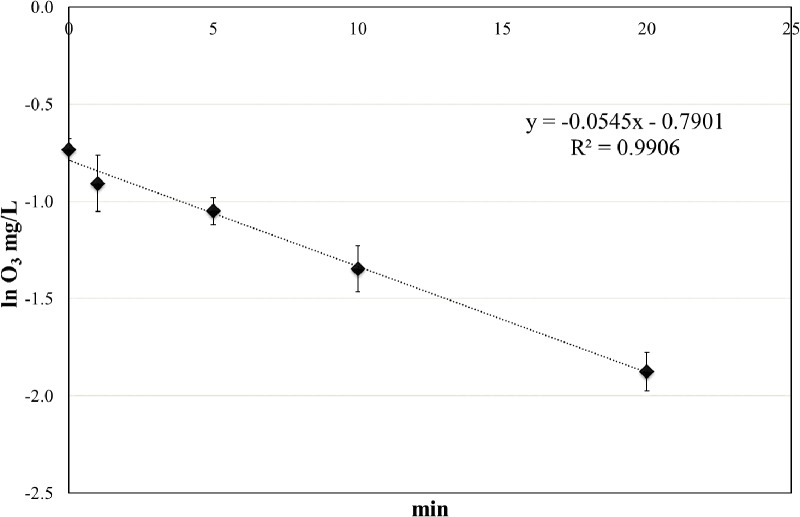
Ozone decay in tap water at room temperature (RT).

The concentration of ozone was measured at different time points (from 0 to 20 min) in closed test-tubes, mimicking the static treatments with aqueous ozone. At time 0, the ozone concentration in water was 0.48 ± 0.06 mg/L, and the concentration progressively decreased reaching mean values of 0.15 mg/L after 20 min. The ozone decay followed a first-order kinetic, with a very high *R*^2^ value (**Figure 2**).

### Treatments With Ozonated Water

**FIGURE 3:**
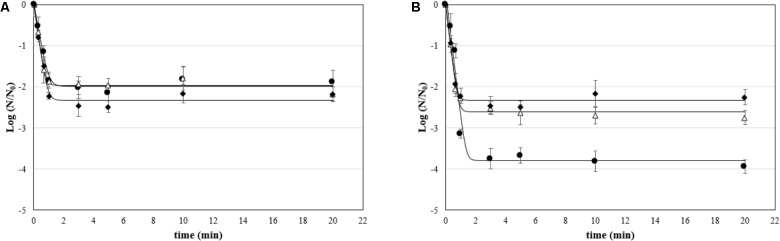
Survival (Log *N*/*N*_0_) of attached cells treated with aqueous ozone in **(A)** static and **(B)** dynamic condition for *P. fluorescens* (•), *S. aureus* (Δ), and *L. monocytogenes* (♦); curves are fitted using the log-linear + tail model.

The effect of ozonated water applied as static and dynamic treatment was evaluated on attached cells and biofilms. The mean viable counts on stainless steel coupons at the beginning of the treatment (untreated controls) were as follows: (i) for attached cells, 5.79 ± 0.18, 4.24 ± 0.12, and 3.88 ± 0.28 Log CFU/cm^2^ for *P. fluorescens*, *S. aureus*, and *L. monocytogenes*, respectively; and (ii) for biofilms, 6.96 ± 0.25, 5.47 ± 0.27, and 5.33 ± 0.18 Log CFU/cm^2^. The counts decreased by increasing the treatment time (see **Supplementary Tables**). The GInaFiT tool was used to estimate the resistance parameters and to identify which model was the best fit for survival curves (Geeraerd et al., 2005). According to our experimental data, the log-linear model with a tail (Geeraerd et al., 2000) was the most appropriate amongst the GInaFiTt list to describe the inactivation kinetics of attached cells treated with ozonated water, as can be seen in **Figure 3**. In fact, the application of this model returned *R*^2^ values significantly higher compared to other models (data not reported). Moreover, relatively low RMSE values were obtained for all trials (**Table 2**). The fitting with a log-linear model with tail allowed to estimate the specific inactivation rate *k*_max_ (1/min), and the Log N_res_, which is the residual population density (Log CFU/cm^2^) at the end of the treatment. Regardless of the microbial species, the inactivation rate of aqueous ozone applied in dynamic conditions was significantly higher than static ones (*p* < 0.05). Moreover, dynamic treatments caused the lowest survival rates on *S. aureus* and *P. fluorescens* attached cells, since the Log *N*_res_ were lower in this condition compared to static one. *L. monocytogenes* attached cells were instead similarly affected in both conditions.

**Table 2:** Kinetic parameters (*k*_max_, specific inactivation constant, and Log *N*_res_, residual population density) and goodness-of-fit parameters of the log-linear + tail model for inactivation by aqueous ozone of attached cells of *P. fluorescens*, *S. aureus*, and *L. monocytogenes*.

	Treatment condition	*k*_max_^∗^	Log *N*_res_^†^	*R*^2^	RMSE
*Pseudomonas fluorescens*	Static	5.09^c^ ± 0.40	3.36^a^ ± 0.07	0.980	0.144
	Dynamic	6.85^a,b^ ± 0.48	1.55^c^ ± 0.17	0.890	0.192
*Staphylococcus aureus*	Static	5.84^c^ ± 0.26	2.00^b^ ± 0.08	0.976	0.161
	Dynamic	7.32^a^ ± 0.17	1.50^c^ ± 0.03	0.934	0.072
*Listeria monocytogenes*	Static	6.69^b^ ± 0.37	1.46^c^ ± 0.08	0.981	0.167
	Dynamic	7.48^a^ ± 0.29	1.63^c^ ± 0.06	0.961	0.157


*^∗^1/min; ^†^CFU/cm^2^. For each column, means followed by different letters are significantly different (p < 0.05).*

**FIGURE 4:**
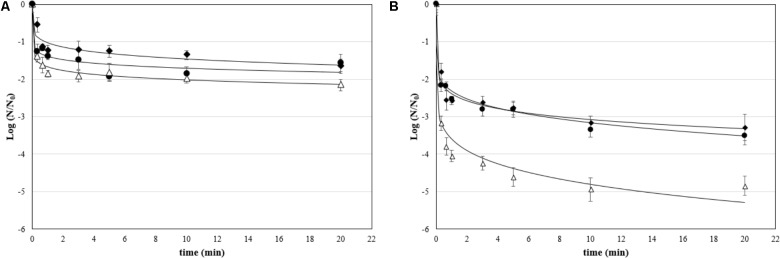
Survival (Log *N*/*N*_0_) of biofilms treated with aqueous ozone in **(A)** static and **(B)** dynamic condition for *P. fluorescens* (•), *S. aureus* (Δ), and *L. monocytogenes* (♦); curves are fitted using the Weibull model.

Unlike attached cells, the most appropriate model to describe the inactivation kinetics of biofilms was the Weibull model (**Figure 4**), which showed a strong fit, as indicated by the high coefficients of determination (*R*^2^ > 0.91) and low RMSE observed for all microbial species (**Table 3**). The fitting with the Weibull model allowed estimating *D*_β_ value, which is the decimal reduction time, i.e., the time for the first decimal reduction of the biofilm viable counts, and ß parameter, which is a shape parameter. Many survival curves exhibit concavity, either downward or upward, and the ß parameter is used to describe this concavity. The β parameters were all less than 1, which indicates the presence of a tail in the curve. As for attached cells, the dynamic treatments of biofilms with aqueous ozone were the most effective, since the *D*_β_ values were lower than static ones, indicating that fewer minutes were required to achieve the 90% inactivation of viable counts. Regardless of microbial species, the antimicrobial effect was stronger in dynamic conditions, since the residual microbial populations after 20 min were lower than in static condition.

**Table 3:** Kinetic parameters (*D*_β_, decimal reduction time; β, shape parameter) of Weibull model for inactivation by aqueous ozone of biofilms of *P. fluorescens*, *S. aureus*, and *L. monocytogenes*.

	Treatment condition	*D*_β_^∗^	β	*R*^2^	RMSE
*Pseudomonas fluorescens*	Static	0.23^b^ ± 0.15	0.09^b^ ± 0.06	0.917	0.271
	Dynamic	0.02^c^ ± 0.00	0.17^a^ ± 0.06	0.952	0.314
*Staphylococcus aureus*	Static	0.02^c^ ± 0.00	0.08^b^ ± 0.02	0.971	0.121
	Dynamic	0.01^c^ ± 0.00	0.11^a,b^ ± 0.03	0.984	0.290
*Listeria monocytogenes*	Static	1.16^a^ ± 0.16	0.15^a^ ± 0.06	0.930	0.213
	Dynamic	0.05^c^ ± 0.00	0.20^a^ ± 0.08	0.919	0.185


*^∗^min. For each column, means followed by different letters are significantly different (p < 0.05).*

### Treatment of Biofilms With Gaseous Ozone

**FIGURE 5:**
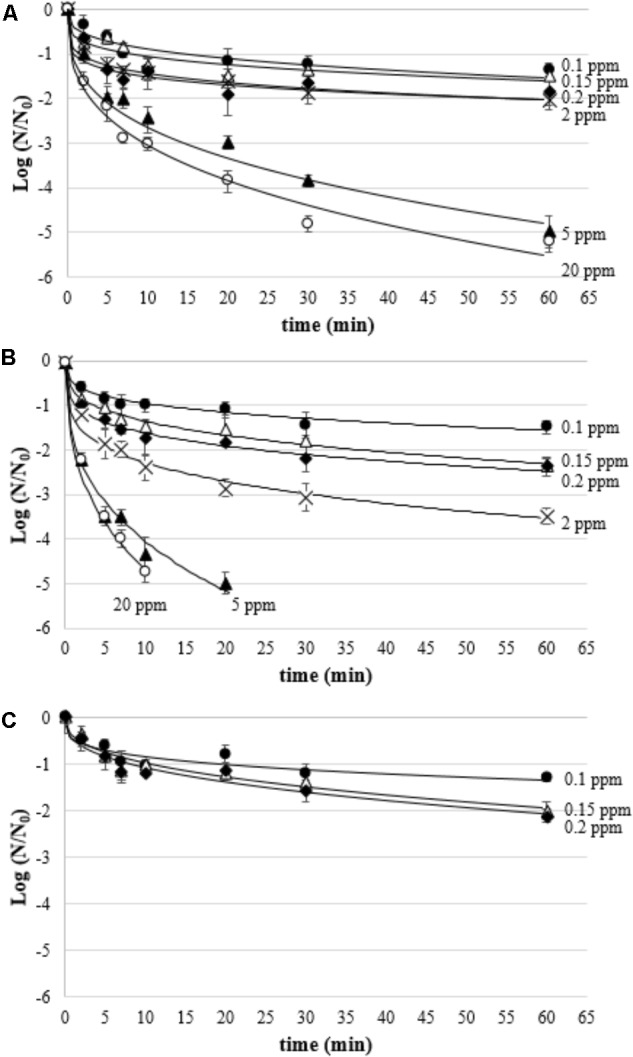
Survival (Log *N*/*N*_0_) of *P. fluorescens* **(A)**, *S. aureus* **(B)**, and *L. monocytogenes* **(C)** biofilms treated with gaseous ozone at 0.1 ppm 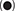, 0.15 ppm (Δ), 0.2 ppm 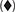, 2 ppm (×), 5 ppm 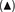, and 20 ppm 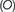; curves are fitted using the Weibull model.

As for biofilms treated with aqueous ozone in dynamic conditions, Weibull model gave the best fit of inactivation kinetics of biofilms treated with gaseous ozone, as evidenced by high *R*^2^ and low RMSE values (**Table 4**). The β parameters were all lower than 1, indicating a tail in the inactivation curve (**Figure 5**). As expected, regardless of the microbial species the *D*_β_ values decreased by increasing the ozone concentration. *L. monocytogenes* showed to be the most resistant species at the lowest ozone concentration (0.1 ppm), having *D*_β_ = 19.74 min compared to *D*_β_ = 12.38 min and *D*_β_ = 10.32 for *P. fluorescens* and *S. aureus*, respectively. Interestingly, starting from 2 ppm the viability of *L. monocytogenes* was undetectable (i.e., <8.3 CFU/cm^2^) even at the 2-min treatments. As for *P. fluorescens*, it was the most resistant species at the highest ozone concentrations, since a residual viability was observed after the treatment at 20 ppm up to 60 min. *S. aureus*, instead, survived the treatment at 20 ppm only for 10 min.

**Table 4:** Kinetic parameters (*D*_β_, decimal reduction time; β, shape parameter) of Weibull model for inactivation by different concentrations of gaseous ozone of biofilms of *P. fluorescens*, *S. aureus*, and *L. monocytogenes*.

	Ozone ppm	*D*_β_^∗^	β	*R*^2^	RMSE
*Pseudomonas fluorescens*	0.1	12.38^b^ ± 2.04	0.29^a,b^ ± 0.04	0.94	0.11
	0.15	7.75^d^ ± 1.55	0.13^b^ ± 0.05	0.94	0.15
	0.2	1.39^f^ ± 0.19	0.18^b^ ± 0.03	0.97	0.12
	2	0.63^g^ ± 0.02	0.16^b^ ± 0.19	0.83	0.43
	5	0.50^g^ ± 0.01	0.33^a^ ± 0.11	0.97	0.18
	20	0.33^g^ ± 0.00	0.33^a^ ± 0.12	0.95	0.24
*Staphylococcus aureus*	0.1	10.32^c^ ± 1.17	0.27^a,b^ ± 0.02	0.93	0.15
	0.15	3.18^e^ ± 0.89	0.19^b^ ± 0.09	0.92	0.12
	0.2	1.10^f^ ± 0.14	0.22^b^ ± 0.01	0.90	0.09
	2	0.30^g^ ± 0.24	0.24^b^ ± 0.12	0.94	0.21
	5	0.18^h^ ± 0.19	0.35^a^ ± 0.22	0.90	0.23
	20	0.15^h^ ± 0.017	0.37^a^ ± 0.14	0.89	0.28
*Listeria monocytogenes*	0.1	19.74^a^ ± 2.98	0.26^a,b^ ± 0.10	0.90	0.17
	0.15	9.87^d^ ± 2.45	0.38^a^ ± 0.06	0.93	0.15
	0.2	8.70^d^ ± 1.18	0.37^a^ ± 0.07	0.95	0.13


*^∗^min. For each column, means followed by different letters are significantly different (p < 0.05).*

## Discussion

The use of ozone and ozonation treatments has proven to be effective as an antimicrobial approach in different contexts, including medical, agricultural, marine, and food (Srikanth et al., 2013; Guo and Wang, 2017). As for the food field, ozone has shown to be a valuable tool for decontaminating different categories of products including dairy, fruit and vegetables, meat and fish, as well as for modifying the textural properties of foods and to inactivate enzymatic activities (Segat et al., 2016; Pinto et al., 2017). Although the role of microbial biofilms as contaminating agents in the food chain is of particular importance, studies on the effectiveness of ozone as an antimicrobial agent against biofilms are currently quite scarce. It is known that the capacity of microorganisms to attach to surfaces and form biofilms is influenced by several factors, which include the chemical–physical characteristics of the material, the incubation conditions, and the microbial strain (Carpentier and Cerf, 1993). All these parameters can also influence the tolerance of microbial cells to the anti-biofilm treatments that are carried out. For this reason, in a preliminary step of the study, the adhesion kinetics of the three microbial species to AISI 304 stainless steel, a widely used material in the food industry for its ease of cleaning and its ability to resist corrosion, have been characterized (Jullien et al., 2003). To account for diversity in growth and survival among different strains of the same species, in this study each trial was carried out using three different strains (one reference and two wild strains) for each microbial species. All three microbial species have shown to adhere very rapidly to stainless steel, probably due to the presence of cell appendages such as pili or fimbriae and flagella, as well as the formation of EPS (Norwood and Gilmour, 1999; Giltner et al., 2006). After 2 h, *P. fluorescens* adhered with a viability of 5.79 Log CFU/cm^2^ and reached its maximum value after just 4 h. The presence of *Pseudomonas* spp. in the food industry is quite risky because they can produce hydrolytic enzymes, pigments, and slimes, which can cause food spoilage, mostly in refrigerated foods (Rajmohan et al., 2002). They can produce exopolymers and attach rapidly to surfaces in the food plants, where they are frequently found. Thus, they can act as reservoirs for the repeated contamination of foods (Lemos et al., 2015). Analogous considerations can be made as for *S. aureus*, which showed to be very fast in adhesion to stainless steel. In fact, the maximum growth rate was similar to that of *P. fluorescens*. However, the maximum cell count after 48 h for *S. aureus* was about 2-Log lower than that of *P. fluorescens*. This result is in agreement with previous studies, which showed that *S. aureus* biofilms are usually less dense than *P. fluorescens* ones (Rossoni and Gaylarde, 2000; Nielsen et al., 2018). Nevertheless, *S. aureus* biofilms could cause cross-contamination via surfaces in the food field. It has to be highlighted that *S. aureus* can cause is food poisoning by producing toxins in food and is a main player in major food poisoning outbreaks worldwide (Bartolomeoli et al., 2009; Ercoli et al., 2017). In the United States, *S. aureus* accounts for more than 240,000 foodborne illnesses per year (Scallan et al., 2011). *L. monocytogenes* showed a slower adhesion kinetic and reached the maximum adhesion after 8 h. Although reaching final count values lower than *P. fluorescens*, these remained quite considerable (5.16 Log CFU/cm^2^). *L. monocytogenes* is considered as one of the key foodborne pathogens of concern, which is responsible for listeriosis in humans, primarily the elderly, pregnant women, and immunocompromised individuals (Ferreira et al., 2014). In the period 2008–2016, there has been an increasing trend of confirmed listeriosis cases, and EFSA reported 2,536 confirmed human cases of listeriosis in 2016. The pathogen was most frequently detected in fish and fishery products, pork meat products, and in soft and semi-soft cheeses made from raw or low-heat-treated milk (EFSA, 2017). This pathogen can successfully colonize food-contact surfaces, and therefore resist for long time in food plants.

The treatments were carried out on attached cells (2-h) and true biofilms (48-h). As for attached cells, several studies indicate that irreversible attachment to surfaces takes from 20 min to a maximum of 4 h (Gilbert et al., 1991; Lundén et al., 2000; Vatanyoopaisarn et al., 2000). As for “biofilm” referring to 48-h cells, similarly, the term “biofilm” is used for cells grown on different materials (including stainless steel) just after 24 h (Di Bonaventura et al., 2008; Williams et al., 2011; Corcoran et al., 2014). The treatments with ozonated water have been applied in two different ways, static and dynamic, that could simulate different uses in the sanitation protocols in the food plants. In the case of treatments under static conditions, these might mimic the dipping of small equipment or disassembled parts of these during brief pauses of the processing operations. Under these conditions, the ozone concentration would be maximum at the beginning of the dipping and would decrease progressively over time due to the decay of the ozone itself. In the case of treatments under dynamic conditions, instead, the surfaces might be treated with a continuous flow of water in which the concentration of ozone remains almost constant since it comes from an ozone generator that operates continuously.

In this study, the inactivation data are reported as values of log (*N*/*N*_0_), that consider both the initial viable counts (*N*_0_, untreated control) and the viable counts after the treatment (*N*). Such way of expressing microbial inactivation is common in the food context (Chang and Craik, 2012). No positive control was prepared, as usual in similar studies (Baumann et al., 2009; Nicholas et al., 2013; de Candia et al., 2015). In fact, in the food field a “gold standard treatment” for biofilm studies doesn’t exist, but a list of measures differently active in terms of active substance, time of treatment, and temperature exists. By using the GInaFiT tool, it was evidenced that the kinetics of attached cells’ inactivation by aqueous ozone were well described by the log-linear + tail model, i.e., a first part in which all cells in the population possess the same resistance, followed by a tail related to the existence of a sub-resistant population. It means that the main inactivation occurred in the first minutes of treatment. Afterward, with increasing treatment time, microbial viability remained constant, not undergoing any significant inactivation. The application of aqueous ozone in the two different modalities had different antimicrobial effects in this study. In particular, the rate of inactivation of the attached cells was higher in the dynamic conditions than static ones regardless of the microbial species. The lower inactivation rates of biofilms in static conditions could be attributed to the fact that the ozone concentration in water progressively decreased over time due to decay. In the treatment conditions tested, in fact, the half-time of ozone was about 10 min. Despite this, at the end of treatment under static conditions (20 min), the log reduction of the microbial load was similar for the three species tested and in the range 2.24–2.43 Log CFU/cm^2^, as inferred by the viabilities of attached cells and the Log *N*_res_ estimated by the model. In the treatments carried out under dynamic conditions, instead, the microorganisms proved to be differently sensitive to treatment. In particular, *L. monocytogenes* was the most resistant species, followed by *S. aureus* and *P. fluorescens*. In the literature, no correlation between the antimicrobial efficacy of ozone and the belonging of microorganisms to the Gram-positive or Gram-negative group has been found. In this study, the higher resistance of *L. monocytogenes* and *S. aureus* species compared to *P. fluorescens* might be related to cellular defense systems against oxidative stress, which are present in both species (Ferreira et al., 2001; Clauditz et al., 2006). As for *P. fluorescens*, the presence of the outer membrane, which can be a cellular shield against other antimicrobial systems (Marino et al., 2001), has not given any defense. Probably, indeed, it has been the first target of the ozone oxidative activity, causing a very high overall loss of viability.

Biofilms treated with ozonated water displayed inactivation kinetics described by the Weibull model with a concave shape. The presence of a concave curve, and not log-linear as in the case of attached cells, could be linked to the fact that the biofilms, being more mature, are made up of a more heterogeneous cell population in terms of age and therefore of physiology (Wimpenny et al., 2000). The existence of a tail clearly indicates that, as in the case of attached cells, a portion of the microbial population is resistant to treatment, due to inherent or acquired resistances during ozonation treatment (Valdramidis et al., 2012). This behavior related to ozonation treatments was also observed by other authors, although not for sessile microorganisms, i.e., in the form of biofilms (Brodowska et al., 2017). The values of the *D*_β_ parameter estimated by the Weibull model in this study indicate that the dynamic treatment was very effective, as it was able to inactivate 90% of the microbial population in the biofilms in a few seconds. Such a treatment applied during downtime during the day immediately after the removal of gross soil, or at the end of the day immediately before the disinfection operation, could contribute to the reduction of the risk of cross-contamination by biofilm.

It is well known that the resistance of biofilms to antimicrobials increases with the age of the biofilm. In fact, adhesion and progressive colonization of surfaces lead to important changes in gene expression and microbial proteomics, which result in an increase in the ability of microbial cells to overcome stress (Fatemi and Frank, 1999; Tremoulet et al., 2002). The data obtained in this study instead show that, when the concentration of ozone in water remains almost constant (under dynamic treatment conditions), biofilms of *S. aureus* and *L. monocytogenes* are more sensitive than attached cells. The reasons for this phenomenon are not clear, even if the mechanisms of resistance seem to be linked to multiple factors and may vary from organism to organism (Patel, 2005). In any case, regardless of the microbial species, the use of aqueous ozone in dynamic condition was able to cause a microbial inactivation of at least 3-Log of biofilm viability after less than 2 min for *S. aureus* and about 8 min for *P. fluorescens* and *L. monocytogenes*. This level of inactivation is considered the minimum inactivation target required for antimicrobials used on biofilms (Mosteller and Bishop, 1993; Rodrigues et al., 2011). These results clearly show that ozonated water can be an effective tool in the control of microbial biofilms in the food industry. It should also be emphasized that this technology is environmental-friendly as the system can be powered simply by tap water. Moreover, the only products generated by the electrolytic cell are ozone, oxygen, and water, so the system does not generate undesired residues (Honda et al., 2013). The use of ozonated water in the food industry is generally obtained through an enrichment of the water with ozone produced by photochemical effect or by the so-called “corona effect” (Tapp and Rice, 2012). Regardless of the ozone generation mode, this must then be dissolved in water through a Venturi system or through appropriate bubble diffusers. A complete system of ozonation of this type consists of several types of equipment, among which oxygen tanks, ozone generator, pumps, valves, and injectors, with consequent high costs both in economic terms and risks for the operators related to the use of high pressures. In the light of these considerations, the possibility of producing ozonated water *in situ* from simple tap water using an electrolytic cell seems to be quite intriguing. Although this requires an extra investment for a minor additional amount of infrastructure in the form of an electrolytic ozone generator, the transportation of potentially dangerous chemicals or the high running costs of an ozone sterilization unit are avoided.

As for aqueous ozone applied in dynamic conditions, the kinetics of inactivation of biofilms through the use of ozone in gaseous form showed a trend described by the Weibull model with tail, indicating that this type of behavior generally characterizes microbial inactivation caused by oxidative processes following ozonation. Gaseous ozone showed to be a less effective antimicrobial than aqueous ozone. In fact, the *D*_β_ values, which measure the time necessary to inactivate 90% of the biofilm viability, were always higher in the case of gaseous ozone, regardless of concentration. The moderate effect exerted by gaseous ozone is strictly due to the mechanism of action of the ozone, which requires the presence of water (Martinelli et al., 2017). Theoretically, increasing the relative humidity could increase the efficiency of gaseous ozone, thus shortening exposure times (Pascual et al., 2007). Despite this, in some conditions, the treatment with gaseous ozone was able to achieve the minimum required efficiency, i.e., 3 Log CFU/cm^2^. In particular, the application of gaseous ozone at a concentration of 5 ppm inactivated 3 Log of microbial biofilms of *P. fluorescens* and *S. aureus* in approximatively 17 and 6 min, respectively. *L. monocytogenes* biofilms showed an unusual behavior, since at the lowest ozone concentrations (up to 0.2 ppm), the viability loss after 60 min was similar to *S. aureus* and *P. fluorescens*, whereas starting from 2 ppm it couldn’t survive (<10 CFU/cm^2^) even after the shortest treatments. Such inactivation levels are particularly interesting as they could be applied to reduce the risk of biofilm in confined environments such as the ripening rooms or warehouses, where biofilms of potentially pathogenic or spoilage microorganisms may occur (Muhterem-Uyar et al., 2015). As for *L. monocytogenes*, it may be present in the biofilm environment of the food industry areas not subject to daily sanitation (Carpentier and Cerf, 1993). The use of ozone in the ripening rooms, carried out in the absence of a product to avoid potential oxidative damage to the lipid component, appears particularly interesting, as these are high humidity environments, which can increase the efficiency of the treatment. Furthermore, this treatment could be carried out during the night in the absence of the personnel in charge, in order to reduce the risks of toxicity related to prolonged exposure to ozone. Such a time interval can be considered sufficient to obtain a satisfactory reduction of the microbial load and at the same time a natural decay of the gaseous ozone, which spontaneously degrades to oxygen (Batakliev et al., 2014). It is also possible to accelerate the ozone decay through specific catalysts or UV lamps in order to further reduce the risks for the operators (Ku et al., 1996; Park et al., 2004).

## Conclusion

The results of this study clearly show that the use of ozone in both aqueous and gaseous form, can have great exploitability in the food industry to reduce the risk related to the presence of microbial biofilms. The use of aqueous ozone can find application in daily practices of equipment sanitizing, both during plant shutdown and at the end of the day. Ozonated water, especially if used in a dynamic condition, can cause a microbial inactivation of at least 3 Log, which is considered a minimum requirement for the antimicrobial substances on biofilms. The fast decay rate of the ozone in these conditions greatly reduces the risk for the operators. Furthermore, the production of ozonized water through an electrolytic cell requires a simple generation system but does not imply further costs of adding ozone to water, as ozone is generated *in situ* directly in tap water. As for gaseous ozone, it is active at higher concentrations, however it could be applied in confined environments (e.g., ripening rooms) overnight, in the absence of the operators to minimize the risk to health. The microorganisms studied in this study are possible causes of spoilage or foodborne diseases, then the use of ozone can become a valid measure in the control of cross-contamination by these bacteria in the food chain. Keeping under control the possible risks for operators and the process parameters, it might be possible to use this strategy as a complement/alternative to conventional sanitization processes, with clear advantages related to environmental impact reduction.

## Author Contributions

MM did conception and design of the work, analysis and interpretation of data, and revising the manuscript. MiM did conception and design of the work, analysis and interpretation of the data, and drafting and revising the manuscript. AB did carrying out of microbiological tests and treatments, and revising the manuscript. NI did design of the work, statistical analysis and interpretation of the data, and revising the manuscript.

## Conflict of Interest Statement

The authors declare that the research was conducted in the absence of any commercial or financial relationships that could be construed as a potential conflict of interest.
