# Supplementary material for: Inactivation of Foodborne Bacteria Biofilms by Aqueous and Gaseous Ozone
Source: Front Microbiol. 2018 Aug 28;9:2024. doi: 10.3389/fmicb.2018.02024 (PMC6120990; doi:10.3389/fmicb.2018.02024)
Supplement: Supplementary file 1 [file Table_1.DOCX]

Inactivation (Log CFU/cm^2^) of attached cells of *P. fluorescens*, *S. aureus* and *L. monocytogenes* by aqueous ozone under static conditions

|  | *P. fluorescens* | *S. aureus* | *L. monocytogenes* |
| --- | --- | --- | --- |
| control | 5.79 ± 0.18 | 4.24 ± 0.21 | 3.88 ± 0.28 |
| 20 s | 5.27 ± 0.22 | 3.58 ± 0.19 | 3.11 ± 0.14 |
| 40 s | 4.65 ± 0.15 | 2.66 ± 0.32 | 2.41 ± 0.11 |
| 1 min | 3.96 ± 0.20 | 2.38 ± 0.17 | 1.66 ± 0.08 |
| 3 min | 3.78 ± 0.26 | 2.31 ± 0.08 | 1.43 ± 0.26 |
| 5 min | 3.65 ± 0.19 | 2.30 ± 0.15 | 1.41 ± 0.15 |
| 10 min | 3.97 ± 0.31 | 2.46 ± 0.29 | 1.72 ± 0.22 |
| 20 min | 3.91 ± 0.28 | 2.07 ± 0.17 | 1.70 ± 0.18 |

Inactivation (Log CFU/cm^2^) of attached cells of *P. fluorescens*, *S. aureus* and *L. monocytogenes* by aqueous ozone under dynamic conditions

|  | *P. fluorescens* | *S. aureus* | *L. monocytogenes* |
| --- | --- | --- | --- |
| control | 5.79 ± 0.18 | 4.24 ± 0.21 | 3.88 ± 0.28 |
| 20 s | 5.27 ± 0.31 | 3.28 ± 0.21 | 2.96 ± 0.09 |
| 40 s | 4.67 ± 0.17 | 2.18 ± 0.17 | 1.96 ± 0.25 |
| 1 min | 2.65 ± 0.11 | 1.98 ± 0.08 | 1.66 ± 0.19 |
| 3 min | 2.05 ± 0.25 | 1.73 ± 0.15 | 1.43 ± 0.22 |
| 5 min | 2.13 ± 0.19 | 1.61 ± 0.29 | 1.41 ± 0.04 |
| 10 min | 1.98 ± 0.25 | 1.54 ± 0.21 | 1.72 ± 0.32 |
| 20 min | 1.85 ± 0.16 | 1.49 ± 0.17 | 1.63 ± 0.19 |

Inactivation (Log CFU/cm^2^) of biofilms of *P. fluorescens*, *S. aureus* and *L. monocytogenes* by aqueous ozone under static conditions

|  | *P. fluorescens* | *S. aureus* | *L. monocytogenes* |
| --- | --- | --- | --- |
| control | 6.96 ± 0.25 | 5.47 ± 0.27 | 5.33 ± 0.18 |
| 20 s | 5.69 ± 0.20 | 4.09 ± 0.17 | 4.78 ± 0.19 |
| 40 s | 5.77 ± 0.05 | 3.84 ± 0.21 | 4.19 ± 0.08 |
| 1 min | 5.58 ± 0.10 | 3.62 ± 0.09 | 4.09 ± 0.15 |
| 3 min | 5.48 ± 0.25 | 3.56 ± 0.15 | 4.12 ± 0.23 |
| 5 min | 5.03 ± 0.12 | 3.66 ± 0.23 | 4.08 ± 0.16 |
| 10 min | 5.11 ± 0.19 | 3.49 ± 0.12 | 3.97 ± 0.12 |
| 20 min | 5.40 ± 0.23 | 3.32 ± 0.15 | 3.68 ± 0.19 |

Inactivation (Log CFU/cm^2^) of biofilms of *P. fluorescens*, *S. aureus* and *L. monocytogenes* by aqueous ozone under dynamic conditions

|  | *P. fluorescens* | *S. aureus* | *L. monocytogenes* |
| --- | --- | --- | --- |
| control | 6.96 ± 0.25 | 5.47 ± 0.27 | 5.33 ± 0.18 |
| 20 s | 4.80 ± 0.17 | 2.30 ± 0.19 | 3.53 ± 0.23 |
| 40 s | 4.77 ± 0.12 | 1.68 ± 0.23 | 2.77 ± 0.26 |
| 1 min | 4.43 ± 0.08 | 1.42 ± 0.15 | 2.76 ± 0.10 |
| 3 min | 4.16 ± 0.19 | 1.23 ± 0.19 | 2.72 ± 0.15 |
| 5 min | 4.15 ± 0.20 | 1.06 ± 0.25 | 2.56 ± 0.19 |
| 10 min | 3.61 ± 0.19 | 1.04 ± 0.31 | 2.17 ± 0.17 |
| 20 min | 3.44 ± 0.24 | 1.00 ± 0.25 | 2.04 ± 0.35 |

Inactivation (Log CFU/cm^2^) of biofilms of *P. fluorescens* by gaseous ozone

|  | 0.1 ppm | 0.15 ppm | 0.2 ppm | 2 ppm | 5 ppm | 20 ppm |
| --- | --- | --- | --- | --- | --- | --- |
| control | 6.96 ± 0.25 | 6.96 ± 0.25 | 6.96 ± 0.25 | 6.96 ± 0.25 | 6.96 ± 0.25 | 6.96 ± 0.25 |
| 2 min | 6.61 ± 0.22 | 6.22 ± 0.19 | 6.31 ± 0.36 | 6.11 ± 0.22 | 5.96 ± 0.19 | 5.36 ± 0.20 |
| 5 min | 6.37 ± 0.15 | 6.29 ± 0.12 | 5.58 ± 0.29 | 5.74 ± 0.15 | 5.01 ± 0.23 | 4.76 ± 0.30 |
| 7 min | 5.96 ± 0.06 | 6.12 ± 0.08 | 5.40 ± 0.20 | 5.61 ± 0.09 | 4.96 ± 0.20 | 4.06 ± 0.09 |
| 10 min | 5.62 ± 0.22 | 5.71 ± 0.19 | 5.54 ± 0.36 | 5.52 ± 0.15 | 4.49 ± 0.30 | 3.96 ± 0.15 |
| 20 min | 5.78 ± 0.31 | 5.48 ± 0.28 | 5.05 ± 0.45 | 5.33 ± 0.29 | 3.97 ± 0.15 | 3.10 ± 0.26 |
| 30 min | 5.73 ± 0.19 | 5.61 ± 0.16 | 5.29 ± 0.33 | 5.10 ± 0.25 | 3.11 ± 0.12 | 2.14 ± 0.18 |
| 60 min | 5.61 ± 0.15 | 5.48 ± 0.12 | 5.11 ± 0.29 | 4.89 ± 0.19 | 1.96 ± 0.36 | 1.73 ± 0.21 |

Inactivation (Log CFU/cm^2^) of biofilms of *S. aureus* by gaseous ozone

|  | 0.1 ppm | 0.15 ppm | 0.2 ppm | 2 ppm | 5 ppm | 20 ppm |
| --- | --- | --- | --- | --- | --- | --- |
| control | 5.47 ± 0.27 | 5.47 ± 0.27 | 5.47 ± 0.27 | 5.47 ± 0.27 | 5.47 ± 0.27 | 5.47 ± 0.27 |
| 2 min | 4.89 ± 0.11 | 4.61 ± 0.21 | 4.47 ± 0.16 | 4.26 ± 0.15 | 3.27 ± 0.15 | 3.27 ± 0.14 |
| 5 min | 4.61 ± 0.17 | 4.45 ± 0.06 | 4.19 ± 0.23 | 3.60 ± 0.32 | 1.99 ± 0.22 | 1.98 ± 0.06 |
| 7 min | 4.47 ± 0.23 | 4.18 ± 0.31 | 3.97 ± 0.14 | 3.48 ± 0.18 | 1.97 ± 0.18 | 1.49 ± 0.19 |
| 10 min | 4.47 ± 0.15 | 4.02 ± 0.15 | 3.75 ± 0.36 | 3.07 ± 0.27 | 1.17 ± 0.36 | 1.05 ± 0.26 |
| 20 min | 4.38 ± 0.18 | 3.97 ± 0.30 | 3.67 ± 0.14 | 2.62 ± 0.19 | 1.02 ± 0.25 | n.d. |
| 30 min | 4.04 ± 0.29 | 3.68 ± 0.11 | 3.30 ± 0.31 | 2.42 ± 0.32 | n.d.* | n.d. |
| 60 min | 3.99 ± 0.15 | 3.11 ± 0.21 | 3.13 ± 0.15 | 2.00 ± 0.18 | n.d. | n.d. |

*n.d., < 8.3 CFU/cm^2^

Inactivation (Log CFU/cm^2^) of biofilms of *L. monocytogenes* by gaseous ozone

|  | 0.1 ppm | 0.15 ppm | 0.2 ppm | 2 ppm | 5 ppm | 20 ppm |
| --- | --- | --- | --- | --- | --- | --- |
| control | 5.33 ± 0.18 | 5.33 ± 0.18 | 5.33 ± 0.18 | 5.33 ± 0.18 | 5.33 ± 0.18 | 5.33 ± 0.18 |
| 2 min | 4.82 ± 0.20 | 4.95 ± 0.19 | 4.87 ± 0.15 | n.d.* | n.d. | n.d. |
| 5 min | 4.70 ± 0.17 | 4.51 ± 0.31 | 4.49 ± 0.12 | n.d. | n.d. | n.d. |
| 7 min | 4.38 ± 0.22 | 4.22 ± 0.22 | 4.18 ± 0.26 | n.d. | n.d. | n.d. |
| 10 min | 4.28 ± 0.15 | 4.33 ± 0.11 | 4.12 ± 0.04 | n.d. | n.d. | n.d. |
| 20 min | 4.53 ± 0.21 | 4.10 ± 0.04 | 4.19 ± 0.19 | n.d. | n.d. | n.d. |
| 30 min | 4.13 ± 0.19 | 3.96 ± 0.12 | 3.76 ± 0.23 | n.d. | n.d. | n.d. |
| 60 min | 4.03 ± 0.08 | 3.34 ± 0.18 | 3.21 ± 0.14 | n.d. | n.d. | n.d. |

*n.d., < 8.3 CFU/cm^2^
